# Outbreak of Amazonian Toxoplasmosis: A One Health Investigation in a Remote Amerindian Community

**DOI:** 10.3389/fcimb.2020.00401

**Published:** 2020-09-11

**Authors:** Romain Blaizot, Cécile Nabet, Laure Laghoe, Benjamin Faivre, Sandie Escotte-Binet, Felix Djossou, Emilie Mosnier, Fanny Henaff, Denis Blanchet, Aurélien Mercier, Marie-Laure Dardé, Isabelle Villena, Magalie Demar

**Affiliations:** ^1^Department of Parasitology-Mycology, Hôpital Andrée Rosemon, Cayenne, French Guiana; ^2^EA 3593, Ecosystèmes Amazoniens et Pathologies Tropicales, Université de Guyane, Cayenne, French Guiana; ^3^Sorbonne Université, INSERM, Institut Pierre-Louis d'Epidémiologie et de Santé Publique, AP-HP, Groupe Hospitalier Pitié-Salpêtrière, Service de Parasitologie-Mycologie, Paris, France; ^4^Department of Pediatrics, Hôpital Andrée Rosemon, Cayenne, French Guiana; ^5^EA 7510 ESCAPE, Université de Reims Champagne-Ardenne, SFR Cap Santé, Reims, France; ^6^Centre National de Référence (CNR) Toxoplasmose/Toxoplasma Biological Resource Center (BRC), Centre Hospitalier-Universitaire de Reims, Reims, France; ^7^Department of Infectious Diseases, Hôpital Andrée Rosemon, Cayenne, French Guiana; ^8^Centres Délocalisés de Prévention et de Soins, Hôpital Andrée Rosemon, Cayenne, French Guiana; ^9^INSERM, IRD, SESSTIM, Sciences Economiques & Sociales de La Santé & Traitement de l'Information Médicale, Aix Marseille University, Marseille, France; ^10^Centre National de Référence (CNR) Toxoplasmose/Toxoplasma Biological Resource Center (BRC), Centre Hospitalier-Universitaire Dupuytren, Limoges, France; ^11^INSERM, Univ. Limoges, CHU Limoges, UMR 1094, Institut d'Epidémiologie et de Neurologie Tropicale, GEIST, Limoges, France

**Keywords:** indigenous, toxoplasmosis, outbreak, rainforest (Amazon forest), parasitology

## Abstract

**Background:** *Toxoplasma gondii* is a parasite of worldwide importance but its burden in indigenous communities remains unclear. In French Guiana, atypical strains of *T. gondii* originating from a complex rainforest cycle involving wild felids have been linked to severe infections in humans. These cases of Amazonian toxoplasmosis are sporadic and outbreaks are rarely described. We report on the investigation of an outbreak of acute toxoplasmosis in a remote Amerindian village. We discuss the causes and consequences of this emergence.

**Methods:** In May 2017, during the rainy season and following an episode of flooding, four simultaneous cases of acute toxoplasmosis were serologically confirmed in two families living the village. Other non-diagnosed cases were then actively screened by a medical team along with epidemiological investigations. Inhabitants from nine households were tested for *T. gondii* antibodies and parasite DNA by PCR when appropriate. Samples of water, cat feces and cat rectal swabs, soil, and meat were tested for *T. gondii* DNA by PCR. Positive PCR samples with sufficient DNA amounts were genotyped using 15 microsatellite markers.

**Results:** Between early May and early July 2017, out of 54 tested inhabitants, 20 cases were serologically confirmed. A fetus infected at gestational week 10 died but other cases were mild. Four patients tested positive for parasite DNA and two identical strains belonging to an atypical genotype could be isolated from unrelated patients. While domestic cats had recently appeared in the vicinity, most families drank water from unsafe sources. Parasite DNA was recovered from one water sample and nine soil samples. Three meat samples tested positive, including wild and industrial meat.

**Conclusions:** The emergence of toxoplasmosis in such a community living in close contact with the Amazon rainforest is probably multifactorial. Sedentary settlements have been built in the last few decades without providing safe water sources, increasing the risk of parasite circulation in cases of dangerous new habits such as cat domestication. Public health actions should be implemented in these communities such as safe water supply, health recommendations, and epidemiological surveillance of acute toxoplasmosis. A “One Health” strategy of research involving medical anthropology, veterinary medicine, and public health needs to be pursued for a better understanding of the transmission routes and the emergence of this zoonosis.

## Introduction

*Toxoplasma gondii* is a ubiquitous parasite that may be transmitted by the consumption of uncooked meat containing viable tissue cysts, or food and water contaminated with oocysts from the feces of infected felids (Hill and Dubey, 2002; Jones and Dubey, 2010). Outbreaks of *T. gondii* have been linked to water contamination (Benenson et al., 1982; Bowie et al., 1997; Isaac-Renton et al., 1998; Aramini et al., 1999; de Moura et al., 2006; Heukelbach et al., 2007; Meireles et al., 2015), exposure to domestic cats (Dubey et al., 2004), consumption of meat from infected animals (Choi et al., 1997), or contaminated vegetables (Ekman et al., 2012). This infection is of special concern in pregnant women and immunosuppressed patients (Hill and Dubey, 2002). In the Amazon, the transmission cycle is complex, involving wild animals, humans living close to the rainforest, and atypical strains. These strains do not belong to the main lineages of *T. gondii* and are responsible for severe symptoms, including in immunocompetent patients. These atypical cases have led to the recent description of the entity called “Amazonian toxoplasmosis” (Dardé et al., 1998; Carme et al., 2002, 2009; Demar et al., 2007, 2012). Autochthonous communities such as Amerindians and Maroon people are particularly at risk, due to their low income, lack of health care access, and the importance of hunting and traditional agriculture. Though severe acute toxoplasmosis has been reported in French Guiana, a few cases have been described in Peru and Brazil (Leal et al., 2007; Nunura et al., 2010; Neves Ede et al., 2011). This infection is likely to be under-diagnosed in many rainforest areas of South America (Carme and Demar-Pierre, 2006).

In French Guiana, only one major *T. gondii* outbreak has been described, in the Maroon community in 2003 (Demar et al., 2007). A unique atypical strain was isolated in five of the 11 patients and was responsible for three deaths (one adult and two congenitally infected fetus or neonate). The high lethality of some atypical strains implies that the emergence of *T. gondii* would represent a special concern. Nevertheless, the burden of *T. gondii* is still poorly documented in these remote areas. Indeed, investigating *T. gondii* outbreaks in remote tropical settings is challenging due to unspecific symptoms, shipping delays, and difficulties in processing analyses. In addition, epidemiological investigations may be complicated by difficulties in interviewing patients about food and cultural habits (Robert-Gangneux and Dardé, 2012), or in logistics. Here we describe the first outbreak of severe acute toxoplasmosis in an Amerindian village of French Guiana and investigate the possible routes for infection, via a One Health approach, testing patients, soil, water, and cats. We discuss the challenges posed by *T. gondii* in traditional communities of tropical areas.

## Methods

In May 2017, during the rainy season and following an episode of flooding (Meteo France, 2017), two adult men were seen in outpatient consultation in the health center of Camopi. One presented diarrhea and vomiting, and was accompanied by his 14-year-old daughter who presented similar symptoms. The other adult presented lymphadenopathy, as did his 12-year-old son. All patients had fever for more than 2 weeks. Given the persistence of symptoms after symptomatic treatment, a suspicion of Amazonian toxoplasmosis was raised and a serology was performed, which confirmed these four cases (positive IgM and IgG).

**Figure 1:**
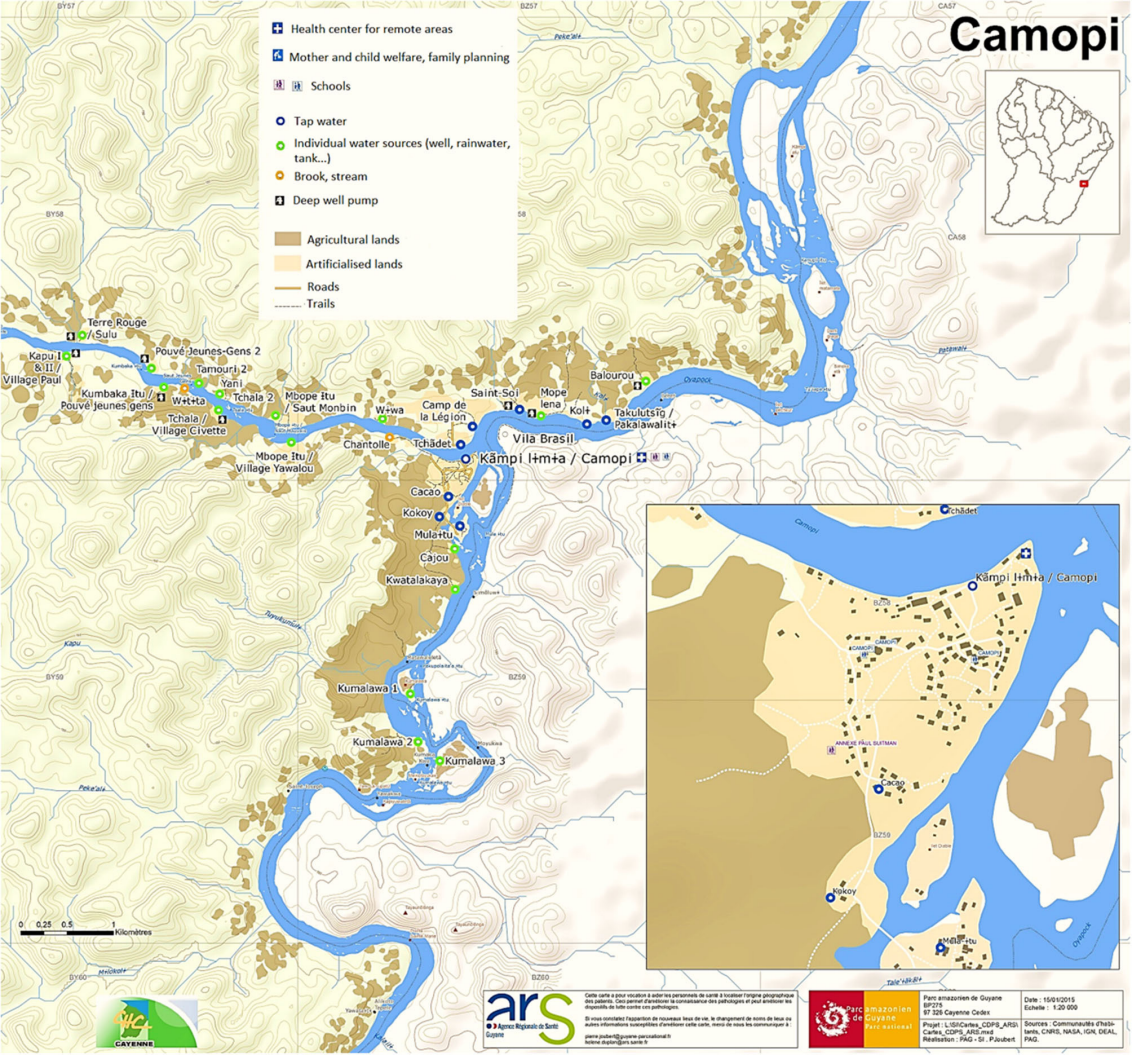
Map of the village of Camopi, provided by the Guiana Amazonian Park (Parc Amazonien de Guyane).

Camopi is an Amerindian village along the Oyapock River, surrounded by tropical rainforest (Figure 1). This remote village of 1,800 inhabitants can be reached from the coastal road after 1 day of canoe. Hunting and fishing are the main productive activities. A demographic transition is under way in this village, as habitations are increasingly sedentary. This transition provides some benefits and new habits such as electrification, drinking water for a few households, and domestication of cats. Teko and Wayampi are the two ethnic groups represented in the village.

After this first epidemiological signal, advice was then given to local physicians to spread information about this possible outbreak throughout the village and encourage villagers with compatible symptoms to get tested. A medical team was dispatched to the village to perform an epidemiological investigation and look for non-diagnosed cases. The investigation team reached the village 1 week after the identification of the first cases. By this time, four new cases had requested a consultation and were serologically confirmed. During the on-field investigation, five other patients spontaneously consulted at the health center and were confirmed by serology.

For each confirmed case, all members of the household were considered contact cases and investigated to look for asymptomatic or mildly symptomatic patients who had not asked for consultation. In case of compatible symptoms and positive serology (positive IgM and positive IgG or IgG seroconversion on two successive blood tests), these contact cases were requalified as confirmed cases (Demar et al., 2007). In each household, individuals were questioned about compatible symptoms and serology was performed for all of them. Individuals were questioned about food practices or other risk factors using questionnaires evaluated during previous investigations (Demar et al., 2007). If present, cat feces were collected and rectal cotton swabs were used to look for carriage of *T. gondii* in cats. Water samples were collected in rainwater cisterns, little streams, and brooks. Meat samples were acquired from the inhabitants and from stores of the Brazilian bank. Soil samples were collected around all houses, gardens, and water sources of infected families. Random soil samples were also collected around the village. Soil sampling was done by electing places in the village where cats had been spotted; sandy or muddy areas close to human habitations; entrances to gardens and orchards; banks of brooks and streams; and surface runoff pathways.

All serum samples were analyzed using the EIA for Toxoplasma-specific immunoglobulin, IgG, and IgM (Abbot Diagnostics). Seropositivity could define either acute toxoplasmosis (IgG+, IgM+) or chronic infection (IgG+, IgM–) and seronegativity meant an absence of infection (IgG–, IgM–), as in previous studies (Demar et al., 2007). When acute toxoplasmosis was serologically confirmed, blood samples and fetal tissues were sent to the National Reference Center (Limoges, France) for *T. gondii* real-time PCR, targeting the AF 487550 gene, strain isolation in mice (two mice for each PCR positive samples), and genotyping with 15 microsatellite markers and strain isolation in mice (Ajzenberg et al., 2010). To analyze the position of the isolated strains, a neighbor-joining tree was reconstructed from the genetic distances Dc (Cavalli-Sforza and Edwards, 1967) using a selection of nine reference strains (Type I, Type II, and Type III) and 42 strains previously isolated from different areas in French Guiana (1000 bootstrap replicates). Unrooted trees were obtained with MEGA version 6 software (Tamura et al., 2013).

Water samples were collected using Envirocheck capsules (Pall Life Sciences, Port Washington, NY, USA). Elution was performed according to previous studies (Lélu et al., 2011; Gotteland et al., 2014). Soil samples went through the first step of treatment with Tween 80 (0.1%) and sucrose 1.20, before a series of centrifugations. Meat samples were homogenized with trypsin and gentamycin before incubation, filtration, and centrifugation according to previous protocols (Mercier et al., 2011). Rectal swabs from cats were incubated overnight at 37°C in PBS-Tween 80 (0.1%). DNA extraction was then performed with a QIAmp®DNA Mini Kit (Qiagen, Courtaboeuf, France) for water, meat samples (from a 200 μl aliquot of the final suspension), and cat rectal swabs and using a FastDNA SPIN kit (MP Biomedical) for soil samples. Amplification of *T. gondii* DNA was assessed targeting the AF 487550 gene (Ajzenberg et al., 2010). Each DNA sample was tested in duplicates. Samples were deemed positive when both wells were positive and undetermined in the case of only one positive well.

PCR analyses were performed between 1 month and 1 year after sample collection, in the Parasitology Laboratories of Cayenne (cats feces and rectal swabs, meat) Limoges (blood samples), and Reims (water and soils).

### Ethics Statement

Methods of this urgent investigation were approved by the relevant Ethics and Public Health authorities (*Agence Régionale de Santé de Guyane, Santé Publique France, Collectivité Territoriale de Guyane, Parc Amazonien de Guyane*). Animal care and use protocol as well as human investigations were approved by the Presidency of the *Collectivité Territoriale de Guyane* (Territorial Collectivity of French Guiana) on behalf of the *Comité Accès et Partages des Avantages* (Committee for Accessing Biodiversity and Sharing Benefits) under the emergency procedure “APA-973-24” (Document N° 340542/2017/PATDDT/DDDT//FB) according to national (article L.331-15-56, Code de l'Environnement; decree 2013-968 approving the charter for the Guiana Amazonian Park) and European (rule 511/2014 of the European Parliament) guidelines. Animal experimentation conducted in Limoges respecting the 3R aspects was approved and accepted by the Ethics Committee for Animal Experimentation n°33 validated by the French Ministry of National Education, Higher Education and Research (Registration numbers: APAFIS#13914-2018030516473189 v2). All adult patients provided a written consent for themselves and their underage children.

## Results

**Figure 2:**
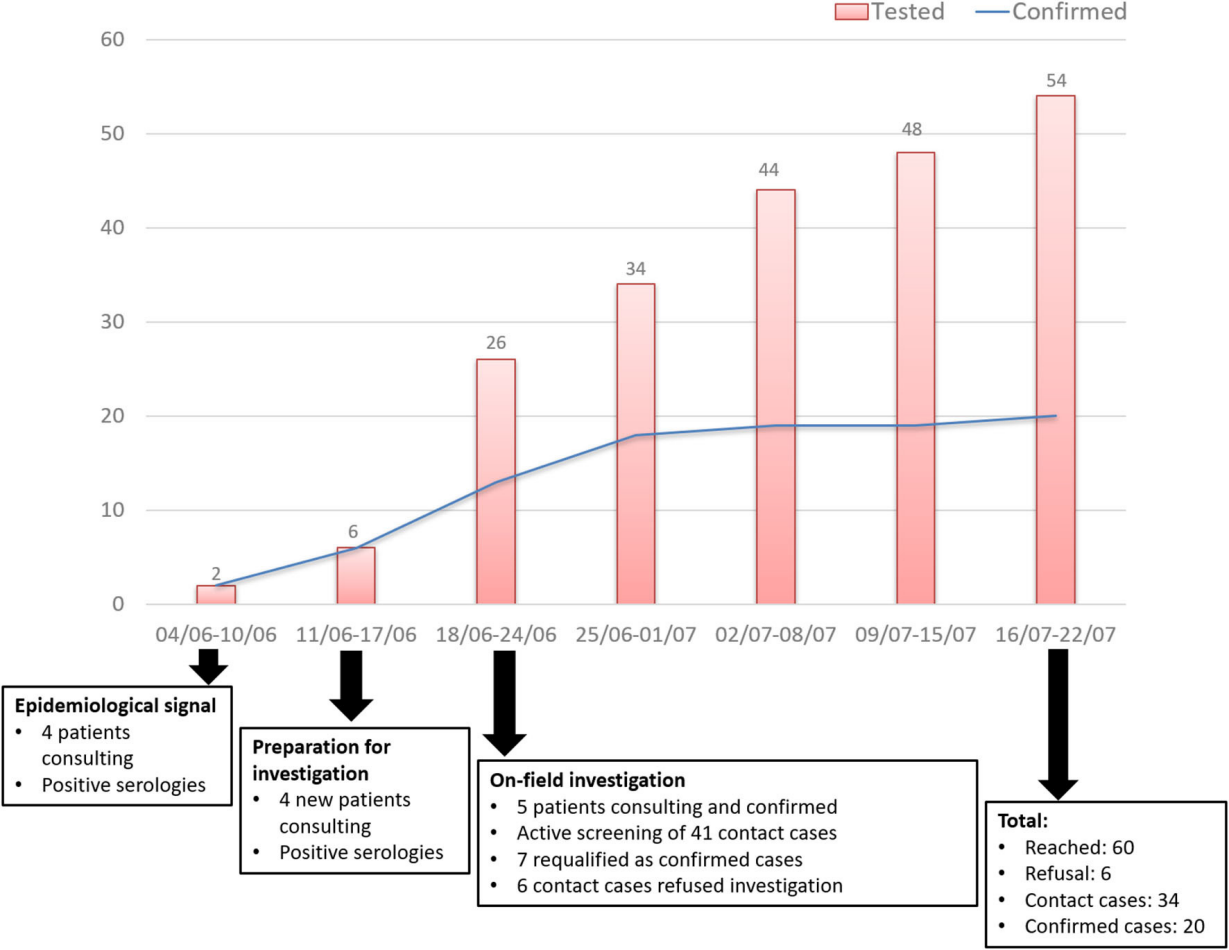
Timeline of the investigation and epidemiological curve of tested and confirmed cases, Camopi toxoplasmosis outbreak, 2017.

During May and June 2017, 60 people were reached, six refused sampling. Twenty cases out of 54 tested inhabitants (37%) (among a population of roughly 1,800) presented a serology compatible with acute toxoplasmosis (positivity of both IgG and IgM anti-*T. gondii*). These 54 people all belonged to households where at least one of the initial patients was diagnosed (Table 1). Some of these contact cases turned out to be confirmed cases after medical examination and blood tests. Confirmed cases were observed in six adults and 14 children belonging to nine different households. All confirmed cases, including asymptomatic ones, presented very high levels of *T. gondii* IgM and IgG, a typical feature of Amazonian toxoplasmosis which favors acute infection rather than chronic infection with residual IgM (Table 2). Other tested inhabitants presented a chronic infection (18.5%) or absence of infection (44.5%). Among the 10 patients with chronic infection, three were more than 50 years old, three were <20 years old, four were aged between 20 and 50. Sex ratio among these patients was 1:1. An epidemiological curve and a chart of the investigation timeline are presented in Figure 2.

**Table 1:** Epidemiological investigation of the 9 infected households, Camopi, French Guiana.

	**No. people living in household**	**Date of first symptoms**	**IgM+ IgG+ confirmed cases**	**IgM– IgG+^d^ contact cases, chronic infection**	**IgM– IgG–^d^ contact cases, absence of infection**	**PCR +^d^ confirmed cases with parasitemia**
Household 1	5	05/05/2017	5 (*n* = 2 adults and *n* = 3 children)	0/5	0/5	0/5
Household 2	6	05/24/2017	2 (*n* = 1 adult and *n* = 1 child)	0/6	4/6	1 (girl, age 14)
Household 3	6	05/17/2017	1 (child)	1/6	4/6	0
Household 4	6^a^	05/31/2017	4 (*n* = 1 adult and *n* = 3 children)	1/5	0/5	2 (pregnant woman and fetus^c^)
Household 5	5^a^	05/29/2017	1 (adult)	0/6	3/6	0
Household 6	6	05/08/2017	2 (children)	1/6	3/6	0
Household 7	6^b^	05/19/2017	3 (*n* = 1 adult and *n* = 2 children)	0/6	1/6	1^c^(boy, age 2)
Household 8	7	06/20/2017	1 (child)	3/7	3/7	0
Household 9	8^b^	06/20/2017	1 (child)	2/6	4/6	0

a *One person refused sampling*.

b *Two persons refused sampling*.

c *Genotyped strain*.

d *IgG+, positive T. gondii IgG antibodies; IgG–, negative T. gondii IgG antibodies; IgM+, positive T. gondii IgM antibodies; IgM–, negative T. gondii IgM antibodies; PCR+, positive T. gondii DNA detection by PCR*.

**Table 2:** Clinical and laboratory features of the 20 cases of acute toxoplasmosis, Camopi, French Guiana.

**Clinical and laboratory features**	**No. cases (%)**
**Clinical signs^a^**	
Asymptomatic	4/20 (20)
Fever	15/20 (75)
Cough/Pneumonia	8/20 (40)
Lymphadenopathy	6/20 (30)
Headache	6/20 (30)
Digestive signs	5/20 (25)
Myalgia	1/20 (5)
Conjunctivitis	1/20 (5)
Skin rash	1/20 (5)
Hepatomegaly	1/20 (5)
**Inpatient care**	5/20 (25)
Adults^b^	3/6 (50)
Children	2/14 (14.3)
**Laboratory disorders**	
Positive *T. gondii* IgG and IgM^c^ Mean levels in IU/mL (min, max): IgG IgM	20/20 (100) 780 (25.2–1814.4) 19.25 (4.1–31.0)
Hyponatremia	6/20 (30)
Hepatic cytolysis	6/20 (30)
Lymphocytosis	3/20 (15)
Elevated creatine kinase	3/20 (15)
High lactate dehydrogenase	3/20 (15)
High C-reactive protein	2/20 (10)
Eosinophilia	2/20 (10)
**Outcome^d^,**	
Complete response^e^	20/20 (100)
Death	0/20 (0)
**Normal Fundoscopy at 6 months**	20/20 (100)

a *Splenomegaly was not detected, but proper examination with an examination table was not possible in traditional houses*.

b *One adult was hospitalized in the Dermatology Department (important skin rash), one in the Infectious Diseases Department, and a pregnant woman was treated in the Obstetrics Department*.

c *Detection threshold: 3IU/mL (IgG) and 0.6 IU/mL (IgM)*.

d *All confirmed cases were treated with sulfamethoxazole (1,600 mg/d) and trimethoprim (320 mg/d) for 21 days. A fetus died at week 19 of pregnancy but was not included in this table of born patients*.

e *Clearance of all symptoms after 21 days of treatment*.

**Figure 3:**
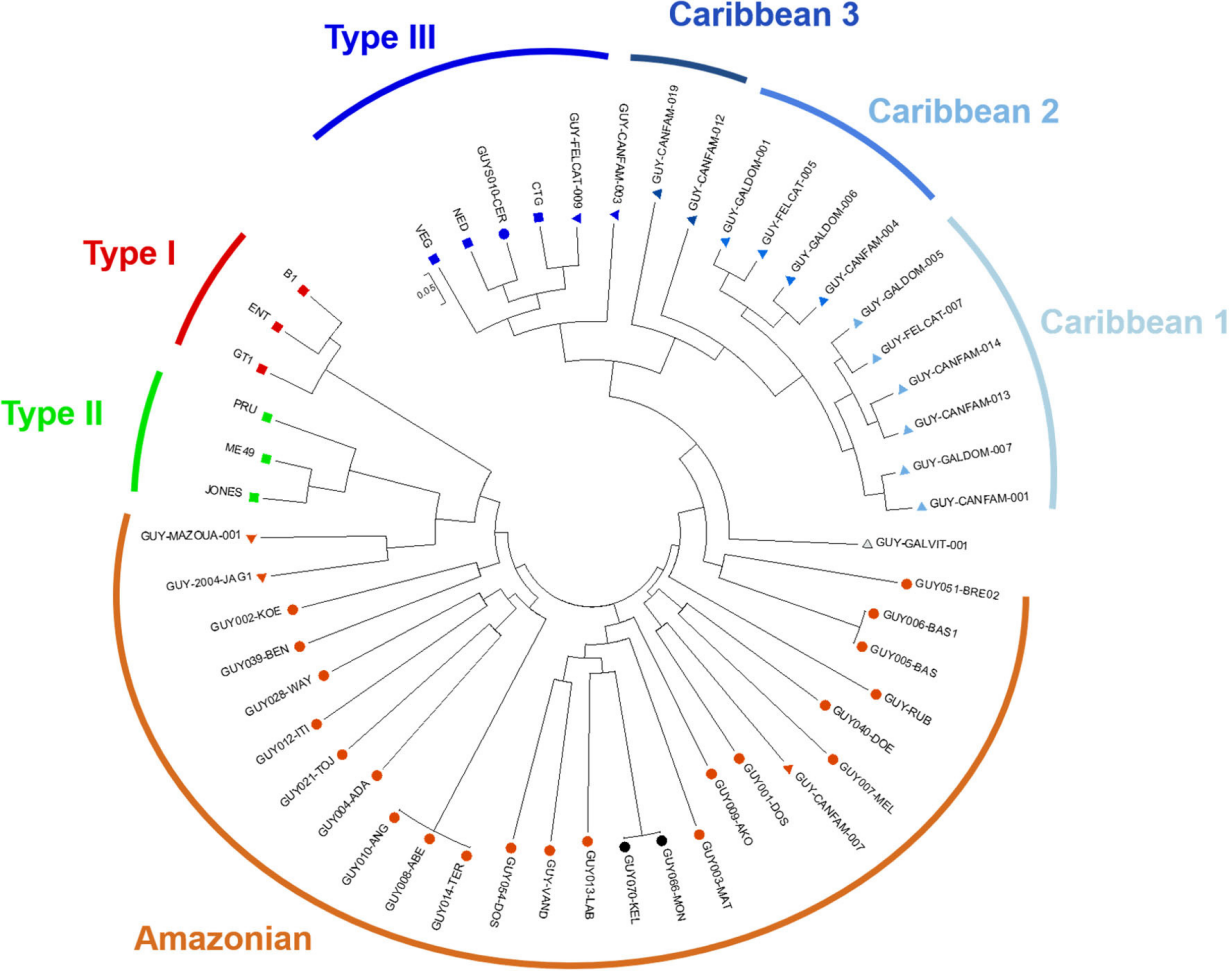
Neighbor-joining clustering of *T. gondii* strains from French Guiana, based on 15 microsatellite markers: circles and triangles correspond, respectively to strains of human and animal origin; black circles correspond to the 2 strains isolated during the Camopi outbreak with 15/15 markers amplified; red, green, and dark blue squares are reference strains of Type I, Type II, and Type III genotype, respectively.

Concerning confirmed cases, mean time to diagnosis was 30 days (median 38 [7–51]), median age of the cohort was 14.5 years old, sex ratio was 3:2. Clinical and biological features of these cases are presented in Table 2. The most frequent clinical symptoms were fever (15 patients, 75%) and cough (eight patients, 40%). The most frequent biological disorders were hyponatremia and hepatic cytolysis (six patients, 30%). Four patients were asymptomatic and diagnosed in the systematic screening of people in contact with cases. Four patients (two adults and two children) were hospitalized due to the risk of clinical worsening, but none of them evolved toward severe pneumonia. Intensive care was never required. A pregnant woman was infected at week 10 of pregnancy and a treatment with spiramycin (rovamycin) 3 g/d was started. At week 19 of pregnancy, fetal cardiac pulsations could no longer be heard and intrauterine death was confirmed by echography. One clonal strain was isolated by PCR in the fetus (liver, brain and cardiac biopsies, and peritoneal fluid) (GUY070-KEL, ID of the Toxoplasma Biological Resource Centre: TgH 18070) and by PCR and mouse bioassay from the blood of a 2-year-old boy with no family relation (GUY066-MON, TgH 18066). These strains clearly belong to the Amazonian genetic group as shown in the divergence tree (Figure 3).

All households shared risk factors for *T. gondii* linked to their traditional way of life: most heads of families were hunters, and children used to eat both game meat and Brazilian chicken. In addition, all families produced and drank homemade caichiri (traditional cassava alcohol) and often shared it with neighbors and relatives. Water was boiled by only one household. Households 7 and 8 used water from deep pumps but could have been infected when drinking caichiri prepared by other families. Other risk factors reflected a new exposure to the parasite due to changes in habits such as domestication of cats and consumption of industrialized meat from stores of Vila Brazil, a small trading post located just on the other side of the river, on the Brazilian bank (Table 3, Figure 1). Epidemiological features of each household are detailed in Table 3. These households are numbered according to their date of diagnosis. Households 1 and 2 harbored the four first cases.

**Table 3:** Risk factors of toxoplasmosis of the 9 infected households, Camopi, French Guiana.

	**Felids around the house**	**Cooking method**	**Water boiling**	**Water sources**	**Homemade products**	**Ethnicity**
Household 1	Domestic cats (*n* = 2)	Boiling buccan	No	Deep well pump Brook	Caichiri Wasai juice	Wayampi
Household 2	No	Boiling buccan stew	No	Rainwater tank Brook	Caichiri Wasai juice Sugar cane juice	Teko
Household 3	Domestic kitten (*n* = 1)	Boiling stewing	Yes	Rainwater tank Brook	Caichiri	Teko
Household 4	Domestic cats (*n* =1 adult and *n* = 1 kitten)	Boiling	Caichiri only	Brook	Caichiri Sugar cane juice	Teko
Household 5	Wild cat (*n* = 1) Domestic cats (*n* = 1 adult and *n* = 1 kitten)	Boiling buccan stew	Cramanioc only	Rainwater tank, brook	Caichiri	Wayampi
Household 6	Puma Jaguarondi Domestic kitten (*n* = 1)	Boiling buccan	Yes	Deep well pump Brook	Caichiri	Wayampi
Household 7	Puma	Boiling	No	Deep well pump	Caichiri	Teko
Household 8	No	Boiling buccan	No	Deep well pump	Caichiri	Wayampi
Household 9	No	Boiling buccan	No	Brook	Caichiri	Teko

*Toxoplasma gondii* was detected by PCR in one water sample out of six from a brook used by a household (Table 4). Parasites were also detected in two out of three pieces of meat, corresponding to frozen chicken from a Brazilian store and a piece of game meat (peccary). Cats rectal swabs were all negative, as were 12 samples of cat feces collected across four households. Two soil samples collected during the investigation were clearly positive, including one collected in front of Household 4 and one random sample from the riverfront of the health center. Seven soil samples were undetermined, five of them around infected households (on the soil of the brook used by Households 5 and 2, and close to homes of Households 4, 5, and 9) and two random samples across the village. MS genotyping was not possible for the PCR positive environmental and meat samples due to an insufficient amount of DNA. Detailed results of environmental samples and meats are presented in Table 4.

**Table 4:** Results of *Toxoplasma gondii* PCR on environmental and meat samples collected during the outbreak investigation, Camopi village, French Guiana.

	**Cat rectal swabs**	**Cat feces**	**Vegetables**	**Meat**	**Water**	**Soil**
Household 1	Negative^a^ (4/4)	Negative (3/3)	–	–	Negative (1 brook)	Negative (3/3)
Household 2	–	–	–	–	Negative (1 brook)	Undetermined (1/1) (Ct 37.8)
Household 3	Negative (2/2)	Negative (2/2)	–	–	–	Negative (3/3)
Household 4	–	Negative (3/3)	Negative (1 fresh cassava)	**Positive (1 peccari, Ct^c^ 36.1)**	–	**Positive (1/5) (Ct 29 and 33.9)** Undetermined (2/5) (Ct 39 35.5) Negative (2/5)
Household 5	Negative (1 kitten, 1 adult)	Negative (4/4)	Negative (1 fried cassava)	–	–	Undetermined (1/1) (Ct 39.4)
Household 6	–^b^	–	–	–	Negative (1 brook)	Negative (2/2)
Household 7	–	–	–	–	–	Negative (2/2)
Household 8	–	–	–	–	Negative (1 deep, 1 well pump)	Negative (2/2)
Household 9	–	–	–	–	**Positive (1 brook, Ct 36.6)**	Undetermined (1/1) (Ct 38.2)
Random soil samples	–	–	–	–	–	**Positive (1/33) (Ct 34.8 and 35.5)** Undetermined (2/33) (Ct 39.3 and 38.9) Negative (30/33)
Brazilian stores	–	–	–	**Positive (2 frozen chicken, Ct 38.1 and 36.8)**	–	–

a *“Positive”, “negative” and “undetermined” corresponds to T. gondii PCR results. Number of tested samples and type of samples are indicated into brackets. Undetermined samples correspond to positivity of only one well over two duplicates*.

b *Not assessed*.

c *Cycle threshold*.

*Bold values correspond to positive PCR*.

## Discussion

This is the largest outbreak of acute toxoplasmosis ever reported in French Guiana, and the first one in an Amerindian community. This epidemiological investigation provided evidence for the spread of *T. gondii* in this remote area, which until now had only reported sporadic cases. We observed several changes in human habits in this community that may have reinforced the exposure to *T. gondii*. The outbreak occurred following an abnormal climatic event with an important episode of flooding and warming. The conjunction of all these factors may have paved the way for an increased parasitic circulation of this zoonotic disease.

Though 16 patients were symptomatic, clinical presentations lacked the respiratory severity that was often reported in Amazonian toxoplasmosis (Carme et al., 2002; Demar et al., 2012). As in previous studies, the involvement of an atypical strain does not seem to be necessarily associated with a poor outcome (Demar et al., 2007; Carme et al., 2009; Blaizot et al., 2018). Indeed, atypical strains are characterized by a high genotypic diversity, especially in the Amazon region, which may correspond to diverse pathogenicity in humans and mice. Moreover, host factors probably play an important role in the severity of Amazonian toxoplasmosis, and genetic susceptibility might be different in this Amerindian population. Indeed, no respiratory involvement was reported in a *T. gondii* outbreak of US military occurring in the Panama jungle (Benenson et al., 1982) nor during the Santa Isabel outbreak in Brazil, which was linked to an atypical strain (de Moura et al., 2006; Vaudaux et al., 2010). Another feature of the Camopi outbreak is the absence of eye involvement, in contrast to what occurs frequently in Amazonian cases of *T. gondii* infection (Carme et al., 2009; Blaizot et al., 2018).

A traditional way of life based on hunting, fishing, gathering, and agriculture seems to be associated with accidental exposures to wild strains of *T. gondii*. Indeed, seroprevalence of *T. gondii* was as low as 18.5% (95% CI [8.1–28.9]) of sampled people during this outbreak. Seroprevalence was even lower (8.3%) amongst the tested population during the Patam outbreak (Demar et al., 2007). In contrast, in a study of three indigenous populations in Brazil, including Wayampi from the Brazilian bank who are ethnically related to their French Guiana counterparts, the authors reported a seroprevalence varying between 50 and 80% (Sobral et al., 2005). An even higher prevalence of specific IgG antibodies was reported in Amerindians of the Venezuelan Amazon (88%) (de la Rosa et al., 1999). These findings show that the level of exposure to *T. gondii* can be very diverse between indigenous populations of Amazonia. In addition, the very low level of exposure in Amerindians leaves them vulnerable to sudden outbreaks, such as the one hereby reported. Even if our sample size is small, it should be noted that we did not report any significant increase of the IgG prevalence with age, in contrast with Brazilian findings (Sobral et al., 2005). We did, however, found an equal sex ratio between male and women with chronic infection, as in the findings of Sobral et al. Men and women are engaged in activities which are frequently at risk of contact with *T. gondii* (hunting for men, cooking for women, agriculture for both genders).

Wild and domestic felids possibly played an important role in spreading oocysts throughout the village. Though seven of the soil samples brought undetermined results, they still bear some significance, due to the poor sensitivity of PCR and the small amount of DNA in soil samples (Lélu et al., 2011; Gotteland et al., 2014). Several families reported a recent domestication of cats in the last 6 months, with multiple births of kittens which were traded or offered as gifts. This recent breeding could have amplified the wild cycle in the direct proximity of dwellings as these felids can mix with their wild counterparts or can hunt infected mammals (Carme et al., 2009; Mercier et al., 2011). Though PCR was negative on all cat feces samples and rectal swabs, these results do not rule out the involvement of domestic or wild felids in this outbreak, due to the low rate of detection for both techniques and the inconstant shedding of oocysts in felids feces (Jones and Dubey, 2010). Though examined cats may not have been shedding cysts at the time of the investigation, they might have shed in the environment several weeks before and contaminated water sources.

A hypothesis of waterborne outbreak can be supported by the detection of *T. gondii* DNA in a water sample from an infected household, consumption of unfiltered water by most inhabitants, sharing of caichiri, and dispersion of cases throughout the village. The 2017 rain season was one of the wettest and hottest ever recorded in French Guiana (Meteo France, 2017), particularly in May, when rainfalls were 32% above normal. As the outbreak happened following this episode of rainfall, oocysts may have contaminated rivers used as water sources. Indeed, it has been shown that oocysts can persist in the soil and can be washed into bodies of water via rain and river flowing (Jones and Dubey, 2010; Lélu et al., 2011). Previous reports in Vancouver and Santa Isabel have highlighted the role of cougars or cats directly infecting water supplies with their feces (Aramini et al., 1999; Vaudaux et al., 2010). This scenario could also have happened in Camopi as most brooks, including the positive one, were not protected by a cap. Consumption of contaminated water has been incriminated in several outbreaks of *T. gondii* in the Americas (Benenson et al., 1982; Pino et al., 2009). Several oocysts-borne outbreaks have been described in Brazil after consumption of contaminated water (Ferreira et al., 2018). Socio-economic and logistical factors such as low access to healthcare, poverty, and lack of water infrastructure have been shown to contribute to oocysts transmission (Ferreira et al., 2018). In peri-urban regions of Brazil, high levels of environmental contamination with oocysts, the presence of a *T. gondii* genetic diversity of strains, a rich wild feline biodiversity in peri-urban, rural, and forested areas, and the proliferation of stray and domestic cats were described as specific factors contributing to a high prevalence of *T. gondii* infections (Shapiro et al., 2019). These features are likely to be found in many endemic areas and were observed in Camopi. A non-archetypal strain of *T. gondii*, isolated from a water supply, was identified as the causative agent of an outbreak in Brazil (Vaudaux et al., 2010). Waterborne infections in previous reports frequently involved hundreds of cases in urban settings due to the contamination of big water reservoirs (Bowie et al., 1997; de Moura et al., 2006) and cases spread over several weeks (Meireles et al., 2015). Similarly, we observed a temporal dispersion of cases from early May to late June. However, the rural environment of the village and the small population along with the absence of a common reservoir for the whole village might explain the relatively small number of cases. Positivity of one water sample out of six was not surprising due to a delay of water collection after the beginning of the outbreak and the small volume used for filtration (10L) to avoid membrane saturation due to the high turbidity. The importance of improving the quality of drinking and irrigation water has been emphasized by a recent review of *T. gondii* outbreaks which highlighted a shift in the epidemiology over the last 20 years, oocysts-mediated outbreaks becoming more frequent in the 2000s (Pinto-Ferreira et al., 2019).

Toxoplasmosis has long been characterized as a food-borne disease and consumption of uncooked game meat has historically been described as a typical cause of Amazonian toxoplasmosis (Carme et al., 2002; Demar et al., 2012). Implication of food in this outbreak was supported by a positive PCR in both game meat and industrial chicken samples. Villagers have changed their habits and now tend to trust frozen meat from Brazilian stores. A broken cold chain could have happened in groceries as we have noticed that this area is not continuously supplied with electricity and power outages are frequent due to the lack of a backup generator. There is no sanitary control in this remote settlement completely isolated from the rest of Brazil. As for cooking, either for industrial or game meat, heating at 60°C for 10 min is necessary to kill all cysts in muscles (El-Nawawi et al., 2008). However, interrogated Amerindians cooked the meat by boiling or buccan. Buccan is a native South American name for a wooden framework on which meat is smoked over a fire. Several men complained of eating meat undercooked by their wives. Fresh vegetables cultivated in rural areas are a possible source of *T. gondii* contamination (Hohweyer et al., 2016) and the ingestion of green vegetables has been associated with an outbreak in Saõ Paulo (Ekman et al., 2012). All households also practiced traditional agriculture in small gardens exposed to felids feces but only two cassava samples could be tested and found negative in PCR.

Amerindian populations of French Guiana have always been exposed to toxoplasmosis due to their practice of hunting and traditional agriculture. However, the recent domestication of cats, which until now were very rare in the village of Camopi, along with the shift from nomadic life to fixed habitations, constitute new risk factors for this disease. The brutal introduction of new life habits in a population not prepared for Western civilization is called an acculturation process. This phenomenon has been previously analyzed as a factor of increased circulation of *T. gondii*, particularly in communities of South America where the presence of domestic cats is coupled with the absence of modern water supply (Chacin-Bonilla et al., 2001; Sobral et al., 2005; Bóia et al., 2008). However, the threat posed by acculturation on the health of Amerindians has never been analyzed to such an extent. Moreover, one should also bear in mind that these findings apply not only to Native Amerindians of South America but also to non-indigenous populations of low economic level and marginal communities in South America (Diaz-Suárez and Estevez, 2009) and many indigenous populations worldwide (Fan et al., 2003; Hotez, 2010; Ngui et al., 2011). Indeed, we report among the Teko and Wayampi people several risk factors such as close contact with cats and dogs, consumption of undercooked game meat, drinking un-boiled and unsafe water, children playing in the dirt, or houses with floors made of mixed soil and sand. Interestingly, many of these epidemiological features have been described in indigenous populations who are over-exposed to toxoplasmosis, such as the Orang Asli of Malaysia (Ngui et al., 2011), or aboriginal populations of Northern Thailand (Fan et al., 2003), and the mountainous areas of Taiwan (Fan et al., 1998). In the same way, in Inuit communities of the Arctic, toxoplasmosis has been associated with consumption of caribou and seal meat, or contaminated water (Hotez, 2010).

Although a clonal atypical strain of *T. gondii* was identified in two unrelated patients in this study and confirmed the epidemic transmission, it did not support a specific transmission route. Thus, the main limitation of our study was the difficulty to isolate and compare the strains from positive human and environmental samples. Isolation of strains is challenging due to the transient presence of the parasite and the difficulty in obtaining a complete genotype due to a low amount of DNA. Another limitation derives from the difficulties of medical interrogation in Amerindian villages despite the presence of a translator. Hunters were often reluctant to give away information on their hunting practices and women who are responsible for cooking were unwilling to answer questions. Finally, the absence of a negative control group did not allow us to perform a real case-control study in order to determine a specific way of contamination.

Our findings suggest that numerous actions should be undertaken to improve the management of toxoplasmosis in remote tropical areas. Eradication of cats in islands has been linked to important benefits in terms of public health (de Wit et al., 2019) and should be contemplated in Amerindian communities where intermingling with wild felids is particularly dangerous, as it can enhance human contact with the wild cycle of *T. gondii*. As recently suggested by Pinto-Ferreira et al. (2019), a greater attention should be paid to the quality of drinking and irrigation water, and to the adoption of recommendations for tracking outbreaks with the aim of eliminating transmission routes, avoiding exposure, or inactivating the parasite before consumption. Another improvement in public health policies would be the development of accurate point-of-care tests for *T. gondii* in isolated areas (Begeman et al., 2017). Indeed, the median time between the first symptoms and diagnosis for each patient was very long in our report (38 days) due to a lack of awareness in local clinicians and due to logistical issues in analyzing blood samples. Point-of-care tests should be improved to detect both IgG and IgM, in order to allow biological confirmation of acute toxoplasmosis, and should be tested with atypical genotypes which do not belong to the main lineages. The burden of acute toxoplasmosis in pregnant women has benefited from some attention, showing that the prevalence during pregnancy can be high in low-income, tropical countries (Rostami et al., 2019). However, one should not forget the possible occurrence of acute toxoplasmosis among men and non-pregnant, immunocompetent women, such as in this outbreak in the village of Camopi. Additionally, one must keep in mind that severe toxoplasmosis might occur in other parts of the world and remain under-diagnosed, even though numerous reports of severe acute toxoplasmosis in South America have led to the description of the “Amazonian toxoplasmosis” entity. Indeed, five cases of severe toxoplasmosis in French travelers returning from West and Central Africa have recently been reported in France (Leroy et al., 2019).

In conclusion, the investigation of this toxoplasmosis outbreak highlighted new life habits among this Amerindian community. Fixed habitations have been built in the last few decades but without providing safe water sources. These sedentary settlements increase the risk of grouped cases, particularly if domestic or wild felids are allowed to come in close contact with habitations. Public health policies should target these indigenous communities, providing safe water supply, health recommendations, and epidemiological surveillance of acute toxoplasmosis. Toxoplasmosis was not listed among a recent review of Neglected Tropical Diseases in the Americas (Hotez et al., 2008). There is consequently no roadmap for its control in remote areas and traditional communities. Future studies should look for possible outbreaks or emerging circulation of *T. gondii* in subtropical areas, including outside South America, in order to assess the exact burden of the disease.

## Data Availability Statement

All datasets generated for this study are included in the article.

## Ethics Statement

The studies involving human participants were reviewed and approved by Collectivité Territoriale de Guyane (Territorial Collectivity of French Guiana) on behalf of the Comité Accès et Partages des Avantages (Committee for Accessing Biodiversity and Sharing Benefits) under the emergency procedure APA-973-24 (Document N° 340542/2017/PATDDT/DDDT//FB). Written informed consent to participate in this study was provided by the participants' legal guardian/next of kin. This animal study was reviewed and approved by Collectivité Territoriale de Guyane (Territorial Collectivity of French Guiana) on behalf of the Comité Accès et Partages des Avantages (Committee for Accessing Biodiversity and Sharing Benefits).

## Author Contributions

RB, FD, EM, and FH: clinical data. LL, SE-B, DB, AM, M-LD, and IV: laboratory analysis. RB, CN, LL, BF, and MD: field investigation. RB and CN: drafting. AM, M-LD, IV, and MD: revising. MD: supervision. All authors contributed to the article and approved the submitted version.

## Conflict of Interest

The authors declare that the research was conducted in the absence of any commercial or financial relationships that could be construed as a potential conflict of interest.

## References

[B1] Ajzenberg D. Collinet F. Mercier A. Vignoles P. Dardé M. L. (2010). Genotyping of *Toxoplasma gondii* isolates with 15 microsatellite markers in a single multiplex PCR assay. J. Clin. Microbiol. 48, 4641–4645. 10.1128/JCM.01152-10 20881166 PMC3008440

[B2] Aramini J. J. Stephen C. Dubey J. P. Engelstoft C. Schwantje H. Ribble C. S. (1999). Potential contamination of drinking water with *Toxoplasma gondii* oocysts. Epidemiol. Infect. 122, 305–315. 10.1017/S0950268899002113 10355797 PMC2809621

[B3] Begeman I. J. Lykins J. Zhou Y. Lai B. S. Levigne P. El Bissati K.. (2017). Point-of-care testing for *Toxoplasma gondii* IgG/IgM using Toxoplasma ICT IgG-IgM test with sera from the United States and implications for developing countries. PLoS Negl. Trop. Dis. 11:e0005670. 10.1371/journal.pntd.0005670 28650970 PMC5501679

[B4] Benenson M. W. Takafuji E. T. Lemon S. M. Greenup R. L. Sulzer A. J. (1982). Oocyst-transmitted toxoplasmosis associated with ingestion of contaminated water. N. Engl. J. Med. 307, 666–669. 10.1056/NEJM198209093071107 7110216

[B5] Blaizot R. Nabet C. Blanchet D. Martin E. Mercier A. Dardé M. L.. (2018). Pediatric Amazonian toxoplasmosis caused by atypical strains in French Guiana, 2002-2017. Pediatr. Infect. Dis. J. 73, 330–331. 10.1016/j.ijid.2018.04.4164 29957729

[B6] Bóia M. N. Carvalho-Costa F. A. Sodré F. C. Pinto G. M. Amendoeira M. R. (2008). Seroprevalence of *Toxoplasma gondii* infection among indian people living in Iauareté, São Gabriel da Cachoeira, Amazonas, Brazil. Rev. Inst. Med. Trop. Saõ Paulo. 50, 17–20. 10.1590/S0036-46652008000100004 18327482

[B7] Bowie W. R. King A. S. Werker D. H. Isaac-Renton J. L. Bell A. Eng S. B.. (1997). Outbreak of toxoplasmosis associated with municipal drinking water. The BC Toxoplasma Investigation Team. Lancet 350, 173–177. 10.1016/S0140-6736(96)11105-3 9250185

[B8] Carme B. Bissuel F. Ajzenberg D. Bouyne R. Aznar C. Demar M.. (2002). Severe acquired toxoplasmosis in immunocompetent adult patients in French Guiana. J. Clin. Microbiol. 40, 4037–4044. 10.1128/JCM.40.11.4037-4044.2002 12409371 PMC139686

[B9] Carme B. Demar M. Ajzenberg D. Dardé M. L. (2009). Severe acquired toxoplasmosis caused by wild cycle of *Toxoplasma gondii*, French Guiana. Emerging Infect. Dis. 15, 656–658. 10.3201/eid1504.081306 19331765 PMC2671434

[B10] Carme B. Demar-Pierre M. (2006). [Toxoplasmosis in French Guiana. Atypical (neo-)tropical features of a cosmopolitan parasitosis]. Med. Trop. 66, 495–503. 17201300

[B11] Cavalli-Sforza L. L. Edwards A. W. F. (1967). Phylogenetic analysis: models and estimation procedures. Evolution. 21, 550–570. 10.1111/j.1558-5646.1967.tb03411.x 28563688

[B12] Chacin-Bonilla L. Sanchez-Chavez Y. Monsalve F. Estevez J. (2001). Seroepidemiology of toxoplasmosis in amerindians from western Venezuela. Am. J. Trop. Med. Hyg. 65, 131–135. 10.4269/ajtmh.2001.65.131 11508387

[B13] Choi W. Y. Nam H. W. Kwak N. H. Huh W. Kim Y. R. Kang M. W.. (1997). Foodborne outbreaks of human toxoplasmosis. J. Infect. Dis. 175, 1280–1282. 10.1086/593702 9129105

[B14] Dardé M. L. Villena I. Pinon J. M. Beguinot I. (1998). Severe toxoplasmosis caused by a *Toxoplasma gondii* strain with a new isoenzyme type acquired in French Guyana. J. Clin. Microbiol. 36:324. 10.1128/JCM.36.1.324-324.1998 9431981 PMC124868

[B15] de la Rosa M. Bolívar J. Pérez H. A. (1999). [*Toxoplasma gondii* infection in Amerindians of Venezuela Amazon]. Medicina 59, 759–762. 10752222

[B16] de Moura L. Bahia-Oliveira L. M. G. Wada M. Y. Jones J. L. Tuboi S. H. Carmo E. H.. (2006). Waterborne toxoplasmosis, Brazil, from field to gene. Emerging Infect. Dis. 12, 326–329. 10.3201/eid1202.041115 16494765 PMC3373086

[B17] de Wit L. A. Croll D. A. Tershy B. Correa D. Luna-Pasten H. Quadri P.. (2019). Potential public health benefits from cat eradications on islands. PLoS Negl. Trop. Dis. 13:e0007040. 10.1371/journal.pntd.0007040 30763304 PMC6392314

[B18] Demar M. Ajzenberg D. Maubon D. Djossou F. Panchoe D. Punwasi W.. (2007). Fatal outbreak of human toxoplasmosis along the Maroni River: epidemiological, clinical, and parasitological aspects. Clin. Infect. Dis. 45:e88–e95. 10.1086/521246 17806043

[B19] Demar M. Hommel D. Djossou F. Peneau C. Boukhari R. Louvel D.. (2012). Acute toxoplasmoses in immunocompetent patients hospitalized in an intensive care unit in French Guiana. Clin. Microbiol. Infect. 18:E221–E231. 10.1111/j.1469-0691.2011.03648.x 21958195

[B20] Diaz-Suárez O. Estevez J. (2009). Seroepidemiology of toxoplasmosis in women of childbearing age from a marginal community of Maracaibo, Venezuela. Rev. Inst. Med. Trop. Saõ Paulo. 51, 13–17. 10.1590/S0036-46652009000100003 19229385

[B21] Dubey J. P. Navarro I. T. Sreekumar C. Dahl E. Freire R. L. Kawabata H. H.. (2004). *Toxoplasma gondii* infections in cats from Paraná, Brazil: seroprevalence, tissue distribution, and biologic and genetic characterization of isolates. J. Parasitol. 90, 721–726. 10.1645/GE-382R 15359466

[B22] Ekman C. C. J. Chiossi MF do V, Meireles, L. R. Andrade Júnior H. F. de Figueiredo W. M. Marciano M. A. M.. (2012). Case-control study of an outbreak of acute toxoplasmosis in an industrial plant in the state of São Paulo, Brazil. Rev. Inst. Med. Trop. Saõ Paulo. 54, 239–244. 10.1590/S0036-46652012000500001 22983285

[B23] El-Nawawi F. A. Tawfik M. A. Shaapan R. M. (2008). Methods for inactivation of *Toxoplasma gondii* cysts in meat and tissues of experimentally infected sheep. Foodborne Pathog. Dis. 5, 687–690. 10.1089/fpd.2007.0060 18681796

[B24] Fan C. K. Liao C. W. Wu M. S. Su K. E. Han B. C. (2003). Seroepidemiology of *Toxoplasma gondii* infection among Chinese aboriginal and Han people residing in mountainous areas of northern Thailand. J. Parasitol. 89, 1239–1242. 10.1645/GE-3215RN 14740918

[B25] Fan C. K. Su K. E. Chung W. C. Tsai Y. J. Chiou H. Y. Lin C. F.. (1998). Seroprevalence of *Toxoplasma gondii* antibodies among Atayal aboriginal people and their hunting dogs in northeastern Taiwan. Jpn. J. Med. Sci. Biol. 51, 35–42. 10.7883/yoken1952.51.35 10211430

[B26] Ferreira F. P. Caldart E. T. Freire R. L. Mitsuka-Breganó R. Freitas F. M. Miura A. C.. (2018). The effect of water source and soil supplementation on parasite contamination in organic vegetable gardens. Rev. Bras. Parasitol. Vet. 27, 327–337. 10.1590/s1984-296120180050 30183998

[B27] Gotteland C. Gilot-Fromont E. Aubert D. Poulle M.-L. Dupuis E. Dardé M.-L.. (2014). Spatial distribution of *Toxoplasma gondii* oocysts in soil in a rural area: Influence of cats and land use. Vet. Parasitol. 205, 629–637. 10.1016/j.vetpar.2014.08.003 25178554

[B28] Heukelbach J. Meyer-Cirkel V. Moura R. C. S. Gomide M. Queiroz J. A. N. Saweljew P.. (2007). Waterborne toxoplasmosis, northeastern Brazil. Emerging Infect. Dis. 13, 287–289. 10.3201/eid1302.060686 17479893 PMC2725844

[B29] Hill D. Dubey J. P. (2002). *Toxoplasma gondii*: transmission, diagnosis and prevention. Clin. Microbiol. Infect. 8, 634–640. 10.1046/j.1469-0691.2002.00485.x 12390281

[B30] Hohweyer J. Cazeaux C. Travaillé E. Languet E. Dumètre A. Aubert D.. (2016). Simultaneous detection of the protozoan parasites Toxoplasma, Cryptosporidium and Giardia in food matrices and their persistence on basil leaves. Food Microbiol. 57, 36–44. 10.1016/j.fm.2016.01.002 27052700

[B31] Hotez P. J. (2010). Neglected infections of poverty among the indigenous peoples of the arctic. PLoS Negl. Trop. Dis. 4:e606. 10.1371/journal.pntd.0000606 20126272 PMC2811175

[B32] Hotez P. J. Bottazzi M. E. Franco-Paredes C. Ault S. K. Periago M. R. (2008). The neglected tropical diseases of Latin America and the Caribbean: a review of disease burden and distribution and a roadmap for control and elimination. PLoS Negl. Trop. Dis. 2:e300. 10.1371/journal.pntd.0000300 18820747 PMC2553488

[B33] Isaac-Renton J. Bowie W. R. King A. Irwin G. S. Ong C. S. Fung C. P.. (1998). Detection of *Toxoplasma gondii* oocysts in drinking water. Appl. Environ. Microbiol. 64, 2278–2280. 10.1128/AEM.64.6.2278-2280.1998 9603850 PMC106314

[B34] Jones J. L. Dubey J. P. (2010). Waterborne toxoplasmosis–recent developments. Exp. Parasitol. 124, 10–25. 10.1016/j.exppara.2009.03.013 19324041

[B35] Leal F. E. Cavazzana C. L. de Andrade H. F. Jr. Galisteo A. Jr. de Mendonça J. S. Kallas E. G. (2007). *Toxoplasma gondii* pneumonia in immunocompetent subjects: case report and review. Clin. Infect. Dis. 44:e62–e66. 10.1086/511871 17304443

[B36] Lélu M. Gilot-Fromont E. Aubert D. Richaume A. Afonso E. Dupuis E.. (2011). Development of a sensitive method for *Toxoplasma gondii* oocyst extraction in soil. Vet. Parasitol. 183, 59–67. 10.1016/j.vetpar.2011.06.018 21764217

[B37] Leroy J. Delhaes L. Houzé S. Loubet P. Yéra H. Rossi B. (2019). La toxoplasmose aiguë rare mais grave chez le voyageur de retour d'Afrique tropicale. Médecine et Maladies Infectieuses 49, S118–S128. 10.1016/j.medmal.2019.04.291

[B38] Meireles L. R. Ekman C. C. J. Andrade H. F. de Luna E. J. A. (2015). Human toxoplasmosis outbreaks and the agent infecting form. Findings from a systematic review. Rev. Inst. Med. Trop. Saõ Paulo. 57, 369–376. 10.1590/S0036-46652015000500001 26603222 PMC4660444

[B39] Mercier A. Ajzenberg D. Devillard S. Demar M. P. de Thoisy B. Bonnabau H.. (2011). Human impact on genetic diversity of *Toxoplasma gondii*: example of the anthropized environment from French Guiana. Infect Genet. Evol. 11, 1378–87. 10.1016/j.meegid.2011.05.003 21600306

[B40] Meteo France (2017). Bulletin Climatique Annuel. Matoury: Meteo France, Climatologie Guyane.

[B41] Neves Ede S. Kropf A. Bueno W. F. Bonna I. C. Curi A. L. Amendoeira M. R.. (2011). Disseminated toxoplasmosis: an atypical presentation in an immunocompetent patient. Trop. Doct. 41, 59–60. 10.1258/td.2010.100228 21062937

[B42] Ngui R. Lim Y. A. Amir N. F. Nissapatorn V. Mahmud R. (2011). Seroprevalence and sources of toxoplasmosis among Orang Asli (indigenous) communities in Peninsular Malaysia. Am. J. Trop. Med. Hyg. 85, 660–666. 10.4269/ajtmh.2011.11-0058 21976569 PMC3183774

[B43] Nunura J. Vásquez T. Endo S. Salazar D. Rodriguez A. Pereyra S.. (2010). Disseminated toxoplasmosis in an immunocompetent patient from Peruvian Amazon. Rev. Inst. Med. Trop. Saõ Paulo. 52, 107–110. 10.1590/S0036-46652010000200008 20464132

[B44] Pino L. E. Salinas J. E. López M. C. (2009). Description of an epidemic outbreak of acute toxoplasmosis in immunocompetent patients from Colombian Armed Forces during jungle operations. Infectio 13, 83–91. 10.1016/S0123-9392(09)70729-5

[B45] Pinto-Ferreira F. Caldart E. T. Pasquali A. K. S. Mitsuka-Breganó R. Freire R. L. Navarro I. T. (2019). Patterns of transmission and sources of infection in outbreaks of human toxoplasmosis. Emerging Infect. Dis. 25, 2177–2182. 10.3201/eid2512.181565 31742524 PMC6874273

[B46] Robert-Gangneux F. Dardé M. L. (2012). Epidemiology of and diagnostic strategies for toxoplasmosis. Clin. Microbiol. Rev. 25, 264–296. 10.1128/CMR.05013-11 22491772 PMC3346298

[B47] Rostami A. Riahi S. M. Contopoulos-Ioannidis D. G. Gamble H. R. Fakhri Y. Shiadeh M. N.. (2019). Acute Toxoplasma infection in pregnant women worldwide: a systematic review and meta-analysis. PLoS Negl. Trop. Dis. 13:e0007807. 10.1371/journal.pntd.0007807 31609966 PMC6822777

[B48] Shapiro K. Bahia-Oliveira L. Dixon B. Dumètre A. de Wit L. A. VanWormer E.. (2019). Environmental transmission of *Toxoplasma gondii*: oocysts in water, soil and food. Food Waterborne Parasitol. 15:e00049. 10.1016/j.fawpar.2019.e00049 32095620 PMC7033973

[B49] Sobral C. A. Amendoeira M. R. Teva A. Patel B. N. Klein C. H. (2005). Seroprevalence of infection with *Toxoplasma gondii* in indigenous Brazilian populations. Am. J. Trop. Med. Hyg. 72, 37–41. 10.4269/ajtmh.2005.72.37 15728865

[B50] Tamura K. Stecher G. Peterson D. Filipski A. Kumar S. (2013). MEGA6: molecular evolutionary GeneticsAnalysis version 6.0. Mol. Biol. Evol. 30, 2725–2729. 10.1093/molbev/mst197 24132122 PMC3840312

[B51] Vaudaux J. D. Muccioli C. James E. R. Silveira C. Magargal S. L. Jung C.. (2010). Identification of an atypical strain of *Toxoplasma gondii* as the cause of a waterborne outbreak of toxoplasmosis in Santa Isabel do Ivai, Brazil. J. Infect. Dis. 202, 1226–1233 10.1086/656397 20836703 PMC5718918

